# Volunteer Medical Service Corps

**Published:** 1918-09

**Authors:** Franklin Martin

**Affiliations:** Member of Advisory Commission and Chairman of General Medical Board, Council of National Defense


### Volunteer Medical Service Corps.

Statement by Dr. Franklin Martin, Member of Advisory Commission and Chairman of General Medical Board, Council of National Defense.

### Foreword.

The Volunteer Medical Service Corps was authorized by the Council of National Defense on January 31, 1918. Under this authorization the membership of the corps consisted of all physicians who because of overage, physical disability, dependents and essential home needs were not eligible for service in the Medical Reserve Corps of the Army or Navy.

### Enlarged Scope of the Organization.

On August 5th the Council of National Defense authorized a change in the scope of the organization and an increase and amplification of its Central Governing Board. Membership in the Corps as now authorized, makes eligible to the Corps every legally qualified physician, including women physicians, holding the degree of Doctor of Medicine from legally chartered medical school, without reference to age or physical disability, provided he or she is not already commissioned in the Government Service. This organization has now the approval of the President as indicated in the following letter:

(COPY)

THE WHITE HOUSE
Washington

12 August, 1918.

My Dear Mr. Martin: I have received your letter of August 5th, laying before me the matured plan for the reorganized Volunteer Medical Service Corps, of which you ask my approval. This work was undertaken by you under the authority of the Council of National Defense; it has had great success in enrolling members of the medical profession throughout the country into a volunteer corps available to supply the needs of the Army, Navy and Public Health Service. In cooperation with the General Medical Board of the Council of National Defense, the strong governing board of the reorganized corps will be able to be of increasing service, and through it the finely trained medical profession of the United States is not only made ready for service in connection with the activities already mentioned, but the important work of the Provost Marshal General's Office and the Red Cross will be aided and the problems of the health of the civilian communities of the United States assured consideration. I am very happy to give my approval to the plans which you have submitted, both because of the usefulness of the Volunteer Medical Service Corps and also because it gives me an opportunity to express to you, and through you to the medical profession, my deep appreciation of the splendid service which the whole profession has rendered to the nation with great enthusiasm from the beginning of the present emergency. The health of the Army and the Navy, the health of the country at large, is due to the cooperation which the public authorities have had from the medical profession; the spirit of sacrifice and service has been everywhere present and the record of the mobilization of the many forces of this great Republic will contain no case of readier response or better service than that which the physicians have rendered.

Cordially and faithfully yours,

(Signed) WOODROW WILSON.

Dr. Franklin Martin,
Advisory Commission,
Council of National Defense.

At a meeting of the Central Governing Board, held on Friday, August 2, it was moved by Dr. Sawyer, seconded by Dr. Martin, that the Central Governing Board shall consist of the present Central Governing Board (excepting Sherk, Bradford, and Brophy) and others as follows:

Surgeon General William C. Gorgas, U. S. A.

Surgeon General William C. Braisted, U. S. N.

Surgeon General Rupert Blue, U. S. P. H. S.

Provost Marshal General E. H. Crowder.

Dr. Franklin Martin, Chairman of Committee on Medicine and Sanitation, Council of National Defense.

Dr. Edward P. Davis, President, Volunteer Medical Service Corps.

Dr. John D. McLean, Vice-President.

Dr. Charles E. Sawyer, Secretary.

Admiral Cary T. Grayson, U. S. N.

Dr. F. F. Simpson.
Dr. Frank Billings.
Dr. H. D. Arnold.
Mr. W. Frank Persons—Red Cross.
Dr. Victor C. Vaughan.
Dr. William H. Welch.
Dr. Robert L. Dickinson, Chief of Staff's Office.
Surgeon R. C. Ramsdell, U. S. N., Chief of Personnel Division.
Colonel James S. Easby-Smith, Executive Officer.
Dr. Joseph Schereschewsky, Assistant Surgeon General (Personnel).
Dr. C. H. Mayo or W. J. Mayo.
Dr. William Duffield Robinson.
Dr. George David Stewart.
Dr. Duncan Eve, Sr.
Dr. Emma Wheat Gillmore.

### General Plan.

The Volunteer Medical Service Corps is exactly what its name indicates. It is a gentleman's agreement on the part of the civilian doctors in the United States who have not yet been honored by commissions in the Army and Navy, and a representative board of governors consisting of officials of the Government associated with lay members of the profession, in which the civilian physician agrees to offer his services to the Government if required and asked to so do by the Governing Board.

It is a method of recording all physicians who are not yet in service and classifying them so that their services when required will be utilized in a manner to inflict as little hardship on the individual as possible. It is a method by which every physician not in uniform will be entitled to wear an insignia which will indicate his willingness to serve his Government.

As more than sixty per cent of the physicians of the country will be utilized in caring for the industries at home and the health of the home people, this large percentage of necessity will be expected to maintain their home status and continue their ordinary professional work.

---

A Failure.—"Did you get acclimated when you went to Cuba?"

"Yes, and by the best doctor I could find, but it didn't take."

